# A review about muscle focal vibration contribution on spasticity recovery

**DOI:** 10.3389/fneur.2025.1579118

**Published:** 2025-06-04

**Authors:** Luigi Fattorini, Vito Enrico Pettorossi, Enrico Marchetti, Angelo Rodio, Guido Maria Filippi

**Affiliations:** ^1^Department of Physiology and Pharmacology, Faculty of Pharmacy and Medicine, Sapienza University of Rome, Rome, Italy; ^2^Department of Human Physiology, University of Perugia, Perugia, Italy; ^3^National Institute for Insurance Against Accidents at Work (INAIL), Rome, Italy; ^4^Department of Human, Social, and Health Sciences, University of Cassino, Cassino, Italy; ^5^Department of Neuroscience, Università Cattolica del Sacro Cuore, Rome, Italy

**Keywords:** hypertonia, proprioception, rehabilitation protocol, stimulus frequency, tonic vibration reflex

## Abstract

**Introduction:**

This review analyses the benefits of focal muscle vibration (FV) in the treatment of spasticity enhancing current understanding and promoting sustained improvements in motor function. Findings could support the selection of optimal FV protocols, guide future research, and provide insights into the mechanisms by which FV may improve motor function in individuals with spasticity.

**Methods:**

A systematic search was conducted using the online databases PubMed, Web of Science, and The Cochrane Library. Including criteria: (a) participants presented with chronic spasticity; (b) the intervention involved the application of localized mechanical vibration; and (c) outcomes included neuromuscular functional parameters. Data extraction was performed independently by four reviewers, using a modified version of the 16-item Downs and Black checklist.

**Results:**

A total of 20 studies were selected, most of which investigated on spasticity following stroke, as well as in conditions such as cerebral palsy, multiple sclerosis, and Minamata syndrome. FV effects were assessed using several methodologies: functional scales, digital analysis and electrophysiological evaluations. After-effects were positive and significant in 19 studies, while one study found non-significant results. In three studies, follow-up durations ranged between 1 and 30 days, and exceeded 1 month in seven. When adequate tests were performed, improvements extended to untreated muscles and involved complex motor behaviors.

**Discussion:**

The after-effects of FV appear to be most relevant and long-lasting when a high-frequency (75–120 Hz), small-amplitude sinusoidal vibrations are repeatedly applied. The observed enduring improvements in complex motor behaviors suggest the involvement of sensory-motor mechanisms. These findings are discussed in the context of previous studies on FV.

## Introduction

In recent years, several journals have reported the positive effect of focally administered mechanical vibration on individual muscles (focal vibration, FV) in improving various motor fitness parameters, including strength, readiness, power, and efficiency (1–6) both healthy and sick. Notably, the after-effects demonstrated prolonged, lasting up to several months. Most of the published articles on FV (2, 7–14) attribute the effects to selective, intense, and prolonged activation of the proprioceptive system, particularly neuromuscular spindle receptors, which induces central changes in the motor system (1–6).

As shown in the recent literature review, persistent positive after-effects seem to be preferably elicited by a vibratory stimulation characterized by high-frequency (70–300 Hz), small-amplitude sinusoidal muscle stretch-shortening, which must be repeated for days (4). Regards the functional effects, FV proprioceptive hyperactivation (4, 13–16) likely acts either directly on the muscle control mechanism, inducing a rearrangement of motor control, or by enhancing proprioceptive discriminative ability and refining the establishment of the spatial reference frame (13, 14, 17–19). Thus, intense and prolonged proprioceptive activation could induce persistent motor improvements, even for complex movements, in the absence of specific motor training (4, 20).

Considering these mechanisms, it is conceivable that FV may enhance motor performance in healthy individuals and support the recovery of mobility in orthopedic or neurological conditions, characterized by motor weakness or paresis or flaccidity. Interestingly, there is also consistent evidence supporting the positive effects of FV in patients experiencing undesired muscle hyperactivity linked to heightened proprioceptive reflex activity, as observed in muscle spasticity (21–40). Spastic hypertonia is a common complication following central nervous system injury, affecting 30–40% of individuals with impaired limb function after stroke (41–44). Certainly, the most prevalent cause of such motor unit hyperactivity is the spasticity following a stroke, but childhood cerebral palsy might also play a role. Spasticity severely limits movements, such as walking, and activities of daily living. Therefore, a systematic review of the literature is essential to evaluate for consistency of the positive effects of FV in the management of spasticity. Although the use of FV in spasticity is endorsed by the American Academy of Physical Medicine and Rehabilitation and the American Society of Neurorehabilitation, appropriate best-practice protocols are not yet well defined (45). Although some reviews have reported the positive effects of FV in the treatment of spasticity, they have not specifically focused on the characteristics of the FV protocols that are most effective in producing substantial and long-lasting positive improvements (46–48). Therefore, the present review aims to: (a) compile the literature in which FV interventions have been administered to patients with muscle spasticity; (b) report FV-induced changes in motor function; (c) identify the most effective intervention parameters in relation to both the extent and duration of functional performance; and (d) assess the compatibility of current findings with a recently proposed theory (4). Collectively, these objectives are intended to advance the knowledge on rehabilitation treatments in pathologies characterized by spasticity.

## Materials and methods

The Preferred Reporting Items for Systematic reviews (PRISMA) guidelines were followed in this review (49).

### Data sources

A systematic literature search was conducted from January 1985 to March 2024 in the online databases PubMed, Web of Science, and The Cochrane Library. Medical Subject Headings (MeSH) of the United States National Library of Medicine (NLM) and search terms were included in Boolean search syntax: (vibration) AND (stroke); (vibration) AND (spasticity); (focal vibration) AND (muscle), (segmental vibration) AND (muscle), (local vibration) AND (muscle). Searching was limited to original studies in English language, human species, and full text availability. Other studies were identified through a manual search for potential articles based on the authors’ knowledge.

### Selection criteria

Two reviewers (LF and GMF) independently extracted data from each study using a structured script. The script included study design, sample characteristics (e.g., sample size and gender), experimental and control group characteristics, outcome measures, and timing of results. Inclusion criteria were decided by the consensus statements between the two reviewers. In cases of disagreement, others reviewers (AR and VEP) were consulted to resolve discrepancies. Inclusions criteria were: (a) participants showed a stable condition of muscle spasticity; (b) the intervention treatment involved localized mechanical vibration; (c) outcomes assessed neuromuscular parameters related to conditional abilities.

### Study eligibility

Studies were excluded if treatment was administered to the whole body (i.e., “no focal”); did not present an original investigation (reviews or proceedings); were not published in English language.

### Assessment of methodological quality

The study quality of each publication was evaluated, by LF, GMF, EM, AR, and VEP, using a 16-item checklist (50). The quality scores were classified as “low” for scores < 50\% or equal; “good” for scores between 51 and 75%; and “excellent” if the score was more than 75%.

According to the modified version of the 16-item Downs and Black checklist, the average quality score was 89.8%. All studies had quality score > 75% (excellent) ranging between 81.25 and 93.75. The inter-rater reliability analysis showed a good coherence between the observers, being 0.92 the kappa value.

## Results

**Figure 1:**
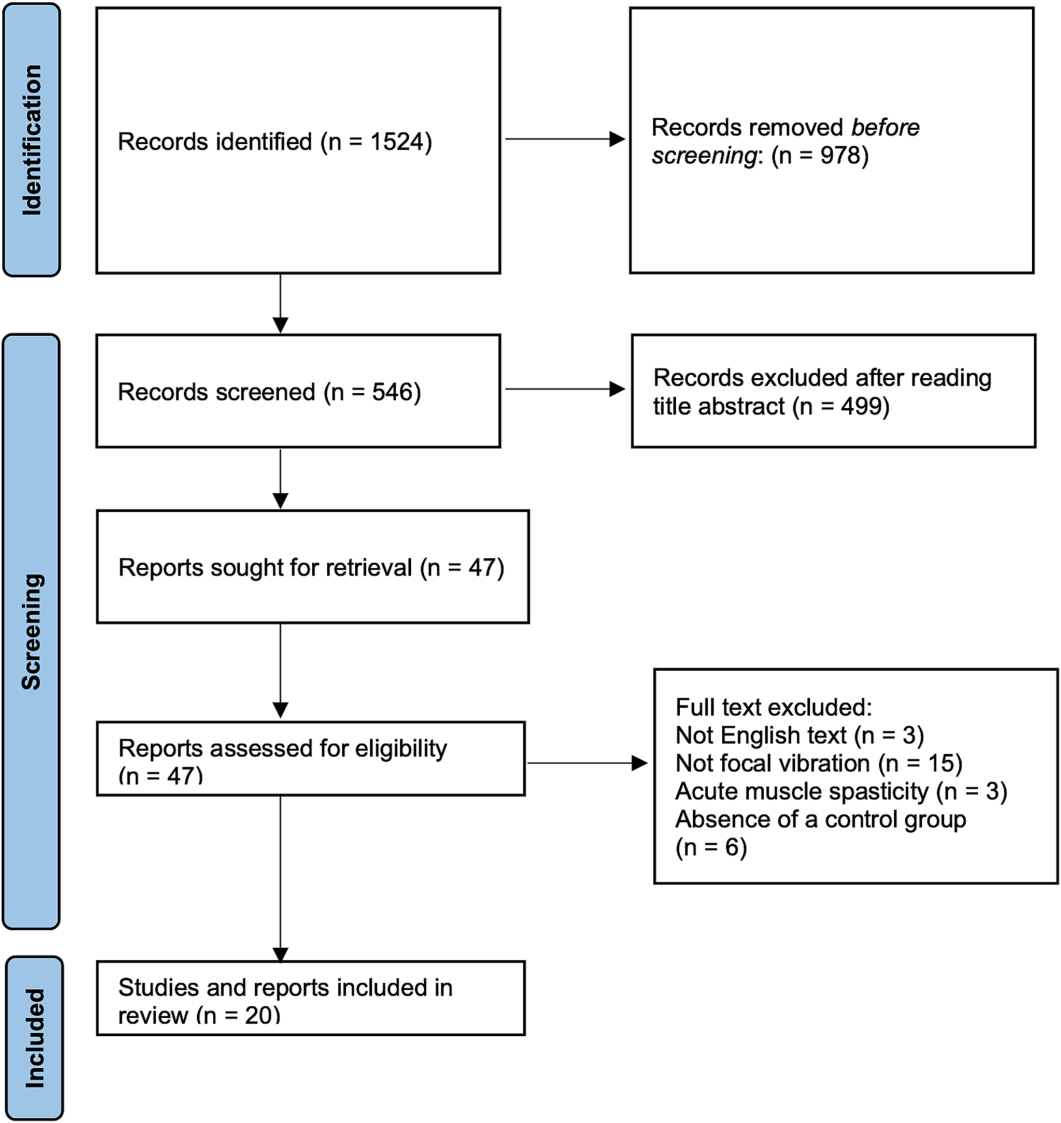
PRISMA workflow of included and excluded articles. PRISMA flow diagram outlining identified studies and exclusion criteria at every level of screening.

Analysis of the literature showed a positive influence of FV on function and motor abilities in neurological diseases characterized by spastic chronic hypertonia. In the present review were selected 20 studies (Figure 1) in which FV was tested on patients with several pathologies (21–40). Eleven articles (22, 25, 27–31, 33, 35–37), out of 20, analysed the effects of FV after stroke, in chronic conditions. In two other studies (23, 26), the participants were children (aged 5–15 years) with symptoms of cerebral palsy. The remaining studies involved motor disabilities associated with multiple sclerosis (21, 24, 34), spinal cord injury (32, 40) and fetal Minamata disease (38, 39). In Table 1 protocols, outcomes, and follow-up are reported.

**Table 1:** Main characteristics of the studies included in this review.

References	Num. Sbjs.	Age (y)	M/F	Disability	Freq (Hz); ampl. (mm); duration (min); repetition	Muscle	Muscle state	Rehab.
Ayvat et al. (21)	33	35	N. R.	Lower limb	50 and 100; 1; 5; 3/w, 8 w	GM	N. R.	Yes
Caliandro et al. (22)	49	59.6	34/15	Upper limb	100; 0.2–0.5; 10; 3/d, 3 d	BB, MP	C	Yes
Camerota et al. (23)	1	5	8/0	Tetraplegia	100; 0.2–0.5; 10; 3/d; 3 d	TS	C	Yes
Camerota et al. (24)	14	48.07	8/6	Lower limb	100; 0.2–0.5; 10; 3/d, 3 d	Q, LPS	C	Yes
Casale et al. (25)	30	64.5	18/12	Upper limb	100; 2; 30; 5/w, 2 w	TB (antagonist)	N. R.	Yes
Celletti et al. (26)	8	6–15	3/5	*Equinus* foot	100; 0.2–0.5; 10, 3/d, 3 d	TS	C	Yes
Celletti et al. (27)	18	49.5	12/6	Upper limb	100; 0.2–0.5; 10; 3/d, 3 d	MP, BB	C	Yes
Costantino et al. (28)	32	61.6	21/11	Upper limb	300; 2; 30; 3/w, 4 w	TB, ECRL, ECRB	C	Yes
Li et al. (29)	10	50.8	5/5	Upper limb	87; 0.28; 3 min; 3/d, 1 d	TS (antagonist)	N. R.	Yes
Liepert and Binder (30)	10	34–72	8/2	Hemiparesis	20; N. R.; 5; 1/d, 1 d	ECR	N. R.	No
Marconi et al. (31)	30	65	17/13	Upper limb	100; 0.2–0.5; 10; 3/d, 3 d	FCR, BB, EDC	C	Yes
Mirecki et al. (32)	8	45–65	8/0	Upper limb extremity	68; N. R.; 0.5; 5/d, 1 d	EWF	N. R.	Yes
Noma et al. (33)	36	61	25/11	Upper limb	91; 1; 5; 1/d, 1 d	BB, FFF	N. R.	No
Paoloni et al. (34)	42	51	15/27	Lower limb	120; 0.01; 30; 3/w, 4 w	RF, GM, GL	N. R.	Yes
Paoloni et al. (35)	22	60.3	13/9	Upper limb	120; 0.01; 30; 5/w, 2 w	BB; FCU	N. R.	Yes
Seim (36)	14	60	9/5	Fingers	67–70; 0.61; 20; 1/d, 1 d	FDS	C	N. R.
Toscano et al. (37)	1	72	1/0	Lower limb	100; 0.2–0.5; 10; 3/d, 4 d	Q, TS, H	C	Yes
Usuki and Tohyama (38)	1	54	1/0	Lower limb	90; N. R.; 15; 2/w, 1 y	PF, H	N. R.	No
Usuki and Tohyama (39)	3	50	3/0	Lower limb	90; N. R.; 15; 1-2/w, 2 y	PF	N. R.	No
Vojinovic et al. (40)	2	65.5	2/0	Upper limb	75; 0.5; 15; 3/w, 2 w	WFE	R	Yes

M/F, Male/Female; s, second; min, minute; d, day; w, week; m, month; y, year; mm, millimeters; N. R., Not Reported; C, Contracted; R, Relaxed; BB, Biceps Brachii; ECR, Extensor Carpi Radialis; ECRB, Extensor Carpi Radialis Brevis; ECRL, Extensor Carpi Radialis Longus; EDC, Extensor Digitorum Communis; EWF, Elbow, Wrist, and Finger Flexor; FCR, Flexor Carpi Radialis; FCU, Flexor Carpi Ulnaris; FDS, Flexor Digitorum Superficialis; FFF, Finger and Forearm Flexor; GL, Gastrocnemius Lateralis; GM, Gastrocnemius Medialis; H, Hamstring; LPS, Lumbar Paraspinal; MP, Minor Pectoral; PF, Plantar Fascia; Q, Quadriceps; RF, Rectus Femoris; TB, Triceps Brachii; TS, Triceps Surae; WFE, Wrist Flexor and Extensor Muscles.

### FV protocols

Two main frequencies ranges of FV can be identified. The first group applied to frequencies between 20 and 70 Hz (21, 30, 32, 36, 38), while the second group used frequencies between 75 and 120 Hz (21–29, 31, 33–35, 37, 39, 40). Three research groups applied only one vibratory session (30, 32, 33), whereas all others repeated the sessions over the course of the week(s). As pointed out in previous reviews (2–4), when vibratory sessions were repeated, two patterns emerged. The first pattern was characterized by FV single sessions distributed across 2–5 days per week (21, 25, 28, 29, 34, 35, 38–40). The second pattern followed a more intense approach, with 3 consecutive days similarly of stimulation, each consisting of 3 sessions per day. Each session lasted 10 min and was separated by 1–2 min of rest (2–4). As shown in Table 1, when amplitude was reported, FV typically ranged between 0.01 and 0.5 mm (22–24, 26, 27, 29, 31, 34–36, 39). In four studies, peak-to-peak amplitude reached up to ≥1 mm (21, 25, 28, 33), while three papers did not report this parameter (30, 38, 39).

### Outcomes

Most of the papers listed in Table 1 used the Modified Ashworth Scale (MAS) (21, 22, 25–28, 31–34, 36, 38–40) and Range of Motion (ROM) as the most common tests (25, 38–40). In addition, also other functional analogue scales (21, 22, 27, 28, 30–32, 34–38, 40), digital analyses (21, 23, 24, 37) and examining electrophysiological correlates of motor effects were adopted to assess changes in complex movements and multi-joint coordination (31, 33, 37).

Regarding spasticity, FV-treated patients, although suffering from different clinical conditions (stroke, cerebral palsy, multiple sclerosis, and Minamata syndrome), achieved significant muscle relaxation, demonstrated by positive improvements in passive joint movements tested by ROM and/or MAS tests (21, 22, 25–28, 31–34, 36, 38–40).

Along with muscle relaxation, a general improvement in voluntary motor activity was reported in almost all studies not only to the treated muscle, but the effects were extended to other muscle districts. In addition, FV treatment resulted in improved movement in several motor tasks of varying degrees of complexity (21–24, 27, 28, 30–32, 36). The effects manifested very early, soon after the end of treatment (21, 23, 28–33, 35, 38, 40).

All but one of the listed authors reported statistically significant improvement in spasticity and voluntary movements (36). However, important differences emerge from the different duration of follow-up. Eight studies tested the effects of FV only immediately after the end of treatment (21, 28–30, 32, 33, 36, 40). This extremely short observation period does not rule out possible persistence of effects, but it does not document it. On the other hand, some studies have reported moderately long follow-up (≥24 h, <1 month) (22, 24, 25, 31, 37–39) and other longer observation intervals, ≥ 1 month (22–24, 26, 27, 34).

Another difference concerns the time interval between the end of FV and the first assessment test. Excluding studies in which follow-up was limited to the end of FV, two studies tested results within 24 h after the end of FV and repeated the test after 2 and 12 weeks (26, 31).

## Discussion

The main findings suggested by the present review are: (a) FV stimulus can improve motor function in patients with muscle spasticity; (b) in several studies, the effects of FV are not only limited to reducing spasticity but also improving motor coordination; and (c) there are important differences among the different studies in terms of the protocols applied, observation period, and positive sequelae. It should be noted that the works listed in this review showed heterogeneity in terms of both the level of functional impairment, due to different pathologies, and the adopted tests.

Interestingly, following FV treatment, patients showed both muscle relaxation and significant improvement in motor coordination when assessed with appropriate tests. A reasonable explanation might be that the reduction of muscle spasticity in the treated muscle, by itself, eliminated important limitations, allowing the adoption of more physiological motor strategies. However, it has been observed that common rehabilitative interventions, aimed at reducing spasticity, do not result in an immediate and proportional improvement of motor gesture (51). This suggests that the early recovery of coordination in complex motor gesture achieved with FV (21–24, 27, 28, 30–32, 36) should be attributed to additional mechanisms elicited by the proprioceptive stimulation, such as those suggested for motor deficit in the presence of reduced mobility and reflexes (4).

### Possible mechanism for explaining the FV effects

Repetitive FV stimulations can be considered as sensorial stimulus that can improve both proprioceptive processing (4, 5) and perception of the spatial reference frames, which can promote both refinement of already known motor behaviors and motor learning (1–4, 15). Regarding the effects of FV on motor adjustments, several authors have suggested that spindle stimulation could improve joint stabilization by acting on the control of joint stiffness (2–4, 7–14, 19). Modulation of joint impedance is a parameter that may result in changes in fatigue, speed, strength, motor task accuracy, and body balance (52, 53). Neurophysiological studies on FV, adopting transcranial magnetic stimulation, seem to propose a convincing background showing a rebalancing of agonist–antagonist activity in the primary motor cortex that can modulate joint impedance (13, 31).

Another interesting feature is the duration of FV effects as also pointed out in previous reviews (2–4). In several articles, reviewed in this study, tests were performed only and immediately at the end of FV stimulation (21, 28–30, 32, 33, 36, 40). Although persistence of the immediately detected significant improvements cannot be ruled out, however, it is not supported by the data. Consequently, this group of study articles cannot offer any insight into this important aspect. However, the remaining papers report long-lasting after-effects that persist, without showing any decay, for weeks and months, suggesting a possible and interesting application role.

### Cortical and spinal plastic changes induced by FV

FV motor positive effects in case of hypotonia can be explained by considering that intense and prolonged proprioceptive activation can potentiate the sensorimotor circuitry excitability through long-term synaptic effects, such as LTP, thus restoring responsiveness of neurons and improving motor performance in the centers of movement planning and execution (3, 4, 13, 15, 20, 31). At the same time, the underlying mechanisms by which FV reduces muscle spasticity remain insufficiently understood. How can same protocol (FV) have positive effects in these opposite conditions?

Marconi and co-workers have shown a long-term increase in FV-induced intracortical inhibition, suggesting an agonist–antagonist rebalance, leading to a remodulation of joint mechanical impedance (13, 31). Such intracortical inhibition correlates with reduced unwanted contractions and improved motor performance in healthy subjects (54–56). However, spasticity is known to be related to hyperexcitability of proprioceptive reflexes. How can the further enhancement of proprioceptive signals by FV lead to both a reduction in muscle spasticity and improvement in motor performance? In the absence of direct experimental evidence, we can only speculate that in the presence of a disinhibition of proprioceptive reflexes due to an imbalance of supraspinal excitatory/inhibitory descending flow, FV could act on this disturbed balance (57). Experimental data report a transient post-FV depression of the stretch reflex, elicited at the spinal cord level by presynaptic or recurrent inhibition (28, 33, 37). These effects occur at the spinal level and, although transient, typically last on the order of minutes, with synaptic control mechanisms appearing to return to baseline within approximately 60 min. Such spinal mechanisms alone are unlikely to account for after-effects that persists for weeks or months (4). However, they could be the trigger for subsequent and persistent cortical rebalancing (31, 57), eventually by interfering with the development of a delayed spasticity (58).

### Comparison between FV protocols

To foster future lines of research and application experiences, it is important to define the determinants of FV and their optimal value related to motor changes and enduring.

FV is commonly applied using sinusoidal muscle shortening-elongation sequences with a small amplitude, it is known to be selectively appropriate for activating proprioceptive muscle afferents (59–61). Likewise, as pointed out in the results, the selected studies showed differences in mechanical stimulation frequency and repetition. Regarding applied stimulation frequencies, two groups of protocols can be identified, one based on 75–120 Hz (21–29, 31, 33–35, 37, 39, 40), while the others adopted stimuli at 20–70 Hz (21, 30, 32, 36, 37). Most of the studies (17 of 20) chose to repeat the application of FV. The repetition of FV, as observed in other reviews, has two different patterns (2–4). A first scheme followed a homogeneous protocol, in which FV applications were performed for 3 consecutive days, with 3 consecutive applications each day, separated by two short rest breaks. A second scheme showed a more distributed and uneven sequence of treatments (i.e., single applications of FV during some days and over one or more weeks).

It should be noted that sustained and documented persistence of after-effects, is associated with the combination of a stimulation frequency of 75–120 Hz with repeated applications. This observation suggests that a repeated stimulation protocol based on a small-amplitude, sinusoidal frequency of 75–120 Hz may contribute to sustained improvements in motor control by inducing central plastic rearrangement (3, 4, 13–15, 20, 62). High-frequency, small-amplitude sinusoidal muscle stretch can drive afferent discharge to various muscle spindles at a correspondingly high rate (59–62). Furthermore, such a low amplitude allows avoidance of the tonic vibration reflex, which could alter the function of central proprioceptive circuits (63). The peak-to-peak shift (0.2–0.5 mm) is in the order of magnitude of the above studies. On the other hand, the effectiveness of much lower displacements, ≈0.01 mm, applied by some studies (34, 35), could be explained by considering the state of muscle spasticity. Isometric muscle contraction facilitates the transmission of mechanical energy and promotes fusimotor activation, that amplifies spindle Ia sensitivity (60, 61, 64). In spasticity, a state of overamplification of the sensitivity of Ia afferents is well known, so that even an extremely low signal could be detected by the neuromuscular spindles, eliciting a high-frequency afferent discharge. Neuronal high frequency activation is known to promote plastic changes in the central nervous system, such as long-term synaptic potentiation (14, 15, 20, 65). In addition, stimulus repetition is a well-known protocol to promote consolidation of plastic rearrangement (65). These observations are supported by a specific experimental study (14) and in tune with previous reviews in which the relationship between FV protocols and beneficial after-effects in healthy individuals and in patients with weak and inadequate muscle contraction has been described (3, 4).

## Conclusion

The methods currently used for spasticity are based on continuous rehabilitative exercise, drug therapy or, sometimes, surgery as last resort (66–68). Present review indicates that FV may be an efficacious additional tool. A highly specific and repeated proprioceptive training based on a sinusoidal waveform in a bounded frequency range, appears to be specifically appropriate to an optimization of motor planning and execution, possibly improving compensatory strategies. Moreover, repeated and preferentially concentrated FV treatment emerge as a more effective protocol in relation to the quality of the effects and their persistence in the follow-up. The above-discussed mechanisms might explain the wide variety of diseases in which FV has induced positive outcomes [see also reviews (2–4)].

Appropriate FV protocols can be used in combination with traditional therapies, or, as suggested in some studies, in patients who exhibit limited or no response to pharmacological treatment (24). Several studies have combined FV with conventional rehabilitation approaches (see Table 1), reporting a booster effect from the combination. However, the current evidence is insufficient to establish a standardized best practice. Several studies have combined FV with traditional rehabilitation (see Table 1), showing a booster effect from the combination. However, the current evidence is insufficient to define a best practice. This represents an important area for further investigation, as future data may support the development of new and more effective interventions.

### Limits

While the reported data provide a more detailed analysis of the effects of FV on spastic symptoms, larger and multicentre studies are warranted to confirm these findings. Moreover, a systematic analysis of these potential interaction between FV and traditional rehabilitation opportune in identifying the best approach to restoring motor function across different spasticity conditions and in different populations.
